# 2283: Brain thermometry to detect neuroinflammation in traumatic brain injury

**DOI:** 10.1017/cts.2017.257

**Published:** 2018-05-10

**Authors:** Kelsey Campbell, Joanne C. Lin, Robert C. Brunner, Thomas A. Novack, Jarred W. Younger

## Abstract

OBJECTIVES/SPECIFIC AIMS: In this pilot study, we are testing a new approach for detecting neuroinflammation in individuals who have sustained a traumatic brain injury (TBI). We hypothesize that many long-term adverse consequences of TBI are driven by abnormal inflammatory processes in the brain that occur secondary to the original neural injury. This inflammation can spread well beyond the damaged tissue and cause profound fatigue, widespread pain, cognitive impairment, and depressed mood. METHODS/STUDY POPULATION: Using a technique based on magnetic resonance spectroscopy, we can obtain precise and accurate temperature measurements throughout the human brain, which may serve as a proxy for neuroinflammation. In this study, we examine 20 men who have sustained a moderate-to-severe TBI and 10 age-matched healthy men without history of TBI. Temperature is assessed on a voxel-by-voxel basis throughout the entire brain. Cognitive ability is measured with the Repeatable Battery for the Assessment of Neuropsychological Status (RBANS). Information on pain, fatigue, and mood is collected through questionnaire. RESULTS/ANTICIPATED RESULTS: We anticipate that (1) average whole-brain temperature will be significantly higher in the TBI group than the healthy control group; (2) severity of (a) pain, (b) fatigue, and (c) mood symptoms will be correlated with brain temperature; and (3) severity of cognitive impairment will be correlated with brain temperature. DISCUSSION/SIGNIFICANCE OF IMPACT: If the hypotheses are confirmed, this tool will fill a need for objective tests of TBI pathology that can be used to improve diagnostic and treatment decisions and predict long-term functioning. This test would be the first completely noninvasive tool for detecting neuroinflammation, and will allow for safe and inexpensive longitudinal testing. Ultimately, we hope this noninvasive scanning technique will accurately track neuroinflammation in TBI, leading to more targeted and effective treatments.

